# SUMOylation of Nuclear γ-Actin by SUMO2 supports DNA Damage Repair against Myocardial Ischemia-Reperfusion Injury

**DOI:** 10.7150/ijbs.74407

**Published:** 2022-07-11

**Authors:** Wei Zhao, Xiuying Zhang, Jia Zhao, Ni Fan, Jianhui Rong

**Affiliations:** 1 School of Chinese Medicine, Li Ka Shing Faculty of Medicine, University of Hong Kong, 10 Sassoon Road, Pokfulam, Hong Kong 999077, China.; 2 Zhujiang Hospital, Southern Medical University, 253 Industrial Road, Guangzhou 51000, Guangdong Province, China.; 3 Shenzhen Institute of Research and Innovation, The University of Hong Kong, Shenzhen 518000, China.

**Keywords:** Myocardial infarction, Ischemia-reperfusion injury, SUMOylation, SUMO2, γ-actin, DNA damage repair

## Abstract

Myocardial infarction triggers oxidative DNA damage, apoptosis and adverse cardiac remodeling in the heart. Small ubiquitin-like modifier (SUMO) proteins mediate post-translational SUMOylation of the cardiac proteins in response to oxidative stress signals. Upregulation of isoform SUMO2 could attenuate myocardial injury via increasing protein SUMOylation. The present study aimed to discover the identity and cardioprotective activities of SUMOylated proteins. A plasmid vector for expressing N-Strep-SUMO2 protein was generated and introduced into H9c2 rat cardiomyocytes. The SUMOylated proteins were isolated with Strep-Tactin^®^ agarose beads and identified by MALDI-TOF-MS technology. As a result, γ-actin was identified from a predominant protein band of ~42 kDa and verified by Western blotting. The roles of SUMO2 and γ-actin SUMOylation were subsequently determined in a mouse model of myocardial infarction induced by ligating left anterior descending coronary artery and H9c2 cells challenged by hypoxia-reoxygenation. *In vitro* lentiviral-mediated SUMO2 expression in H9c2 cells were used to explore the role of SUMOylation of γ-actin. SUMOylation of γ-actin by SUMO2 was proven to be a new cardioprotective mechanism from the following aspects: 1) SUMO2 overexpression reduced the number of TUNEL positive cells, the levels of 8-OHdG and p-γ-H2ax while promoted the nuclear deposition of γ-actin in mouse model and H9c2 cell model of myocardial infarction; 2) SUMO-2 silencing decreased the levels of nuclear γ-actin and SUMOylation while exacerbated DNA damage; 3) Mutated γ-actin (K^68^R/K^284^R) void of SUMOylation sites failed to protect cardiomyocytes against hypoxia-reoxygenation challenge. The present study suggested that SUMO2 upregulation promoted DNA damage repair and attenuated myocardial injury via increasing SUMOylation of γ-actin in the cell nucleus.

## Introduction

Myocardial infarction (MI), commonly known as heart attack, has recently emerged as a leading cause of human death and disabilities worldwide 1, 2. Sequential ischemia and reperfusion not only cause myocardial necrosis, but also induce the overproduction of reactive oxygen species and the intensive release of inflammatory and fibrotic factors, leading to ventricular remodeling 3, 4. Reactive oxygen species oxidize proteins, DNA, lipids and other biomacromolecules 4, 5. Oxidative DNA damage underlies the pathogenesis of various cardiac diseases, including atrial fibrillation and ischemic heart disease 5. The existing interventions mainly restore the blood flow and improve the symptoms while few strategies alleviate myocardial ischemia reperfusion injury (MIRI)^5^, ^6^. Interestingly, oxidative DNA damage may be attenuated by antioxidants, nicotinamide adenine dinucleotide (NAD^+^) boosters and enzymes involved in oxidative DNA repair processes 5. Nevertheless, the proteins that regulate cardiac functions and DNA damage repair are tightly regulated by post-translational modifications (PTMs) including acetylation, methylation, ubiquitinylation and sumoylation 7, 8. Thus, PTMs may be an important tagert for the therapy of myocardial infarction.

SUMOylation describes that a family of small ubiquitin-like modifier (SUMO) proteins, namely, SUMO1-5, form covalent attachment via its C-terminal carboxylic group (-COOH) to the specific lysine residues in a variety of protein substrates to affect the functions of various proteins in cellular metabolism, DNA repair, transcription, cell division and other cellular processes^9^-^11^. SUMO proteins are highly similar in the primary structure, specifically, 48% sequence identity between SUMO1 and SUMO2/3, 97% identity between SUMO2 and SUMO3, 85% identity between SUMO4 and SUMO2/3 and mutual homology between SUMO5 and SUMO1^12^-^14^. Five isoforms are known to display distinct expression patterns and evident functional heterogeneity, suggesting that SUMO proteins may exhibit overlapping *in vivo* functions 15, 16. SUMOylation not only affects cardiac function and development but also promotes the adaptation of the heart to different pathological stimuli 14, 17, 18. SUMO1 and SUMO2/3 play key roles in the SUMOylation of the transcriptional factors (e.g., GATA4-6, MEF2, NKX2-5, SRF, TBX2, and TBX5) to dictate cardiac development and the signaling proteins (e.g., ERK5, NF-κB, *p53*, PKC) to determine the progression of atherosclerosis 19. Under the physiological conditions, SUMOylation and deSUMOylation form dynamic balance to coordinate the regulation of cardiac development, metabolism and stress adaptation while the pathological stimulations disrupt such balance, leading to cardiac dysfunction and myocardial injury 14, 20. It was demonstrated that SUMO overexpression enhanced cardiac functions in mice with heart failure and increased contractility in isolated cardiomyocytes 21, 22. In contrast, the deficiency in SUMO-modification of several substrates appeared to be inextricably linked to dilated cardiomyopathy 23. Nevertheless, SUMO isoforms may mediate the SUMOylation of different proteins to alleviate or exacerbate myocardial damage 20, 24, 25. The components in the SUMOylation pathway underwent dramatically changes in ischemic heart disease 26. These studies suggest that SUMOylation may be an important therapeutic target for drug discovery against myocardial infarction.

The SUMOylation of proteins is regulated by multiple signaling pathways including mitogen-activated protein kinase (MEK), extracellular signal-regulated kinase (ERK) and phosphatidylinositol 3-kinase (PI3K) 27-29. Synthetic SUMOylation inhibitors (e.g., TAK-981) were evaluated for activating IFN1 signaling pathway and controlling DNA damage response and gene transcription 30, 31. Natural products ginkgolic acid and astragaloside IV were also evaluated for targeting SUMOylation of proteins in animal models of MI and cell culture systems 32, 33. Along this line, we previously demonstrated that plant-derived C-glycosylated isoflavone puerarin exhibited effective cardioprotective potential in a mouse model of myocardial infarction via enhancing SUMO2-mediated SUMOylation without affecting SUMO1 expression 34. However, little is known about the protein substrates that are SUMOylated by SUMO2 in myocardial infarction. Herein, we prepared a N-Strep-SUMO2 fusion protein for affinity purification of SUMOylated proteins from cardiomyocytes. We further investigated the roles of SUMOylated proteins in the myocardial infarction.

## Material and Methods

### Antibodies and Reagents

Dulbecco's modified Eagle's medium (DMEM), fetal bovine serum, penicillin, and streptomycin were purchased from Thermo Fisher Scientific (Waltham, MA, USA). Antibodies against SUMO2 and Alexa Fluor 488/647-conjugated goat anti-IgG secondary antibodies were purchased from Invitrogen (Carlsbad, CA, USA). Antibodies against 8-OHdG and p-γ-H2ax were purchased from Santa Cruz Biotechnology Inc (Dallas, Texas, USA), whereas antibody against GAPDH was purchased from Cell Signaling Technology (Boston, MA, USA). ML-792 was purchased from Selleck Chemicals (Houston, TX, USA). Unless otherwise indicated, other biochemical reagents were purchased from Sigma-Aldrich (St. Louis, MO, USA).

### H9c2 cells culture and hypoxia/reoxygenation (H/R) model

Rat embryonic cardiomyocyte H9c2 cells were obtained from the American Type Culture Collection (Manassas, VA, USA) and cultured in high glucose DMEM containing 10% FBS, 100 U/mL penicillin, and 100 μg/mL streptomycin at 37 °C in a humidified incubator containing 5% CO_2_. H9c2 cells were washed twice with PBS and incubated in glucose-free DMEM with or without the drug for hypoxia-reoxygenation treatment. The cells were transferred to an Eppendorf Galaxy 48R hypoxia chamber (Hamburg, Germany) under the conditions of 0.1% O_2_ and 5% CO_2_ at 37 °C for 3 h. After removing glucose-free DMEM, the cells were incubated in high-glucose DMEM containing 10% FBS and transferred to the regular cell culture incubator under the conditions of 5% CO_2_ and 95% air for 24 h 25. For drug treatment, the cells were grown to 70-80% confluence in the complete growth medium and treated with ML-792 (1 μM). In contrast, the control cells were treated with an equal amount of dimethyl sulfoxide (DMSO) under the same conditions.

### Identification of SUMO2-bound proteins

Full-length murine SUMO2 cDNA was inserted into mammalian expression vector pcDNA3.1(+) plasmid to yield pcDNA3.1-N-Strep-SUMO2, encoding the N-Strep- SUMO2 fusion protein. The plasmid pcDNA3.1-N-Strep-SUMO2 was introduced into rat embryonic cardiomyocyte cell line H9c2 by electroporation at 220 V, 500 μF, single pulse. The SUMOylated proteins were isolated by the Strep-Tactin XT 4Flow Starter Kit (2-5998-000) from IBA Lifescience (Göttingen, Germany) according to the manufacturer's instruction. In brief, the transfected cells were cultured at 37 °C, 5% CO2 for 48 h, harvested and resuspended in Buffer W and sonicated. After centrifugation at 13000 rpm for 15 min, the supernatant was loaded to the Strep-Tactin XT column, washed and eluted. The eluate was concentrated with Amicon® Ultra Centrifugal filter unit from Merck KGaA (Darmstadt, Germany). The concentrate was separated by 10% SDS-PAGE gel and stained with Coomassie blue G-250 dye. The major protein bands were recovered and subjected to proteomic identification at the Centre for PanorOmic Sciences (CPOS), the University of Hong Kong.

### Animal husbandry

The experimental procedures were approved by the Committee on the Use of Live Animals in Teaching and Research of the University of Hong Kong (CULATR 4899-18). Adult male C57BL/6N mice (10-12 weeks, 25-30 g) were supplied by the Centre for Comparative Medicine Research, the University of Hong Kong, and housed in a humidity- and temperature-controlled environment on a 12 h light-dark cycle and offered free access to the standard laboratory animal diet and drinking water.

### Mouse model of myocardial ischemia-reperfusion injury (MIRI)

For animal experiments, the sample size was calculated using the formula: Sample size = 2* SD2 (Zα/2 + Zβ) *2 /d2. It was previously demonstrated that the mean myocardial infarction area was 16.32% for mice model and 6.85% for sham groups while SD is 6.45% 34. When the level of significance at 5% and power of study at 80% were selected for two tailed unpaired t -test, the sample size = 2* (6.45)2* (1.96 + 0.842) *2 / (9.47)2 = 5.2. Thus, 6 animals were used for each experimental group. Mice were randomly divided into two groups, Sham and MIRI. For MIRI group, mice were anesthetized by *i.p*. injection of 100 mg/kg ketamine and 10 mg/kg xylazine under a mouse volume-control ventilator (55-7040) from Harvard Apparatus (Holliston, MA, USA). The heart was exposed following thoracotomy between the 3rd and 4th intercostal space, and the left main coronary artery was subsequently ligated with a 6-0 silk suture. After 45 min ischemia, the suture was loosened to allow reperfusion for functional recovery over 24 h. The mice were monitored for consciousness and pain response in a warm and comfortable nesting cage. In case of surgical pain, mice were treated by *i.p.* injection of 0.1 mg/kg buprenorphine every 12 h for at last three days. For Sham group, mice were not subjected to the ligation of the left main coronary artery.

### Western blot analysis

The protein levels were detected by Western blot analysis as previously described 35. In brief, heart tissues and H9c2 cells were lysed with RIPA buffer containing 1× protease inhibitor cocktail and centrifuged at 13,000 rpm at 4 °C for 20 min. The supernatants were recovered and determined for the protein concentrations by Bio-Rad protein assay dye reagent concentrate (Hercules, CA, USA). Proteins (60 μg for tissue samples, 30 μg for cells) were separated with 10% or 12% SDS-polyacrylamide gel electrophoresis and transferred onto polyvinylidene fluoride membranes (0.45 µm). The membranes were blocked in 5% BSA buffer for 2 h, probed with primary antibodies at 4 °C overnight and incubated with HRP-conjugated secondary antibodies at room temperature for another 1 h. The blots were visualized with Amersham ECT™ detection reagent from GE Healthcare (Uppsala, Sweden). The blots were quantified by NIH ImageJ software (http://imagej.nih.gov).

### Co-immunoprecipitation

Co-immunoprecipitation experiments were performed as previously described 36. Briefly, H9c2 cells were transfected with indicated constructs. Forty-eight hours later, the cells were lysed in RIPA buffer (Sigma-Aldrich, Cat#R0278) and protease inhibitor cocktail (Roche, Cat# 4693159001). Lysates were incubated with the anti-SUMO2 or γ-actin antibodies and washed by the lysis buffer. Bead-bound proteins were resolved by SDS-PAGE and detected by immunoblotting using indicated antibodies.

### Confocal immunofluorescence analysis

Following the treatment, H9c2 cells were fixed in 4% paraformaldehyde, permeabilized with 0.5% Triton X-100 for 30 min and blocked in 5% normal goat serum at room temperature for 2 h. The cells were probed with anti-γ-actin or anti-8-OHdG antibodies at 4 °C overnight. The bound antibodies were detected with Alexa Fluor 488/647 anti-mouse IgG secondary antibody at room temperature for 2 h. After three washes with PBS, the cell nuclei were stained with DAPI for 5 min. Immunofluorescence co-localization analysis was performed on LSM 980 laser scanning confocal microscope from Carl Zeiss (Jena, Germany) according to the previous publication 37. Confocal immunofluorescence images were analyzed using NIH ImageJ software (http://imagej.nih.gov), while Pearson's colocalization coefficient was calculated for all colocalization analyses. Fluorescence intensity was quantitated for each wavelength using the 'plot profile' function and subsequently plotted by Sigmaplot.

### Terminal deoxynucleotidyl transferase dUTP nick end labeling (TUNEL) assay

DNA fragmentation was detected by TUNEL assay with the TUNEL assay kit from Vazyme Biotech (Nanjing, China) as previously described 38. Briefly, the cell monolayers or 8 mm frozen tissue sections were mounted on glass coverslips, washed with PBS and incubated with proteinase K solution at room temperature for 3 min. After the incubation with equilibration buffer for 30 min, the slides were rinsed and incubated with the TUNEL reaction mixture in a humidified chamber at 37 °C for 60 min. After washing with PBS, the sections were stained with DAPI for 10 min, washed with PBS and visualized under a fluorescence microscope.

### Lentivirus-mediated overexpression or silencing of SUMO2 in H9c2 cells

Lentiviral expression vector plasmids of pCDH-CMV-SUMO2 and pLKO.1-CMV-SUMO2 respectively for rat SUMO2-cDNA and shRNA with the sequence of 5′ - CCGGGACTGAGAACAACGATCATATCTCGAGATATGATCGTTGTTCTCAGTCTTTTTGAATT-3′ were purchased from IGE Biotechnology Ltd (Guangzhou, China). For the preparation of lentiral particles, 293FT cells were pre-seeded, co-transfected with the combo of lentivirus packaging vectors (i.e., pRSV-Rev, pMDLg/Prre, pMD2.G) and transfer plasmid with either of pCDH-CMV-SUMO2 and pLKO.1-CMV-SUMO2 plasmid. The transfected cells were cultured at 37 °C for 48 h to allow the production of lentiviral particles. For the overexpression and silencing of SUMO2, H9c2 cells were subsequently infected with the corresponding lentiviral particles over 48 h. The stable clones were selected in cell culture medium containing 1 μg/ml of puromycin.

### Mutation of SUMOylation sites (i.e., K^68^ and K^284^) in γ-actin

For expressing γ-actin-GFP fusion protein, fulllength γ-actin cDNA was cloned into mammalian expression vector pEGFP-N3 from Clontech. Briefly, rat ACTG cDNA was amplified by PCR technique using the following primers: ACTG-F: 5′-GTACGAATTCGCCGATCGCAATGGAAGA-3′, ACTG-R: 5′-GTACGGATCCGAAGCATTTGCGGTGGACA-3′. The PCR product was digested with the restriction enzymes EcoRⅠ and BamHⅠ and cloned into the pre-cut pEGFP-N3 vector. The SUMOylation sites (i.e., K^68^ and K^284^) in γ-actin were subsequently mutated with Mut Express Ⅱ Fast Mutagenesis Kit V2 from Vazyme Biotech (Shanghai, China) to yield plasmid construct pEGFP-ACTG (K^68^R /K^284^R). Point mutation was performed using oligonucleotide sequences as follows: pEGFP-rACTG-K68R-Forward: 5′-ACCCTGAGGTACCCTATTGAGCACGGCATTGT-3′, pEGFP-rACTG-K68R-Reverse: 5′-ATAGGGTACCTCAGGGTCAGAATACCCCTCTTG-3′, pEGFP-rACTG-K284R-Forward: 5′-CATCATGAGGTGTGATGTGGACATCCGCAAAG-3′, pEGFP-rACTG-K284R-Reverse: 5′-CATCACACCTCATGATGGAGTTGAAGGTGGTC-3′. For the overexpression of γ-actin (K^68^R/K^284^R), H9c2 cells were subsequently transfected with the corresponding plasmid over 48 h. The stable clones were selected in cell culture medium containing 1 μg/ml of puromycin.

### Statistical Analysis

The data were presented as mean ± SD from at least three independent experiments and analyzed by one- or two-way ANOVA (analysis of variance), followed by Dunnett's test or LSD's test, using GraphPad Prism software (San Diego, CA, USA). A *p*-value of < 0.05 was considered statistically significant.

## Results

### γ-actin was identified as a potential substrate for SUMOylation by SUMO2

**Figure 1:**
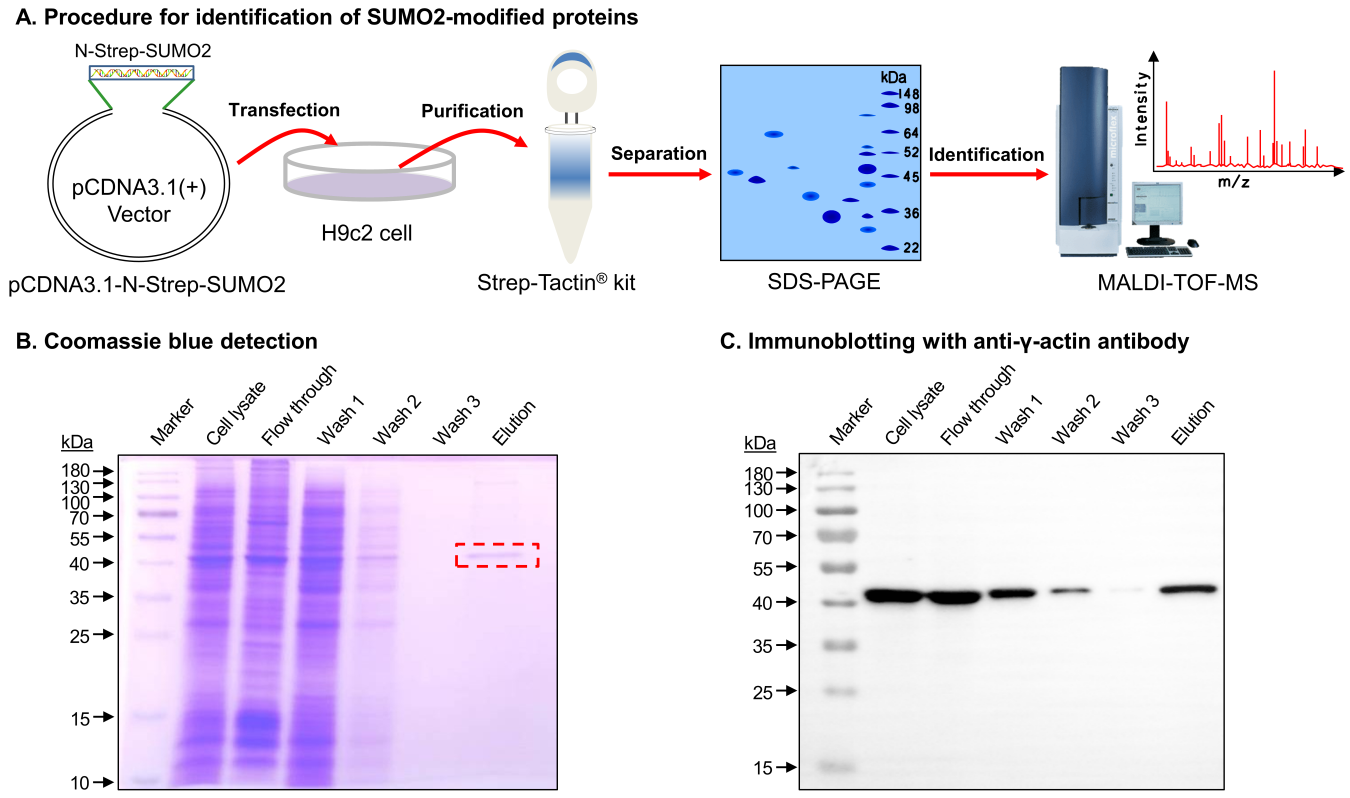
** Isolation and verification of the SUMOylated proteins from cardiomyocyte H9c2 cells. (A)** Scheme illustrating the isolation and identification of the SUMOylated proteins from cardiomyocyte H9c2 cells. pcDNA3.1-N-Strep SUMO2 plasmid was introduced into H9C2 cells for expressing N-Strep-SUMO2 fusion protein. The SUMOylated proteins were pulled down, resolved by SDS-PAGE and analyzed by MALDI-TOF-MS technology. **(B)** Separation and Coomassie blue staining. The protein fractions were resolved by gel electrophoresis and detected by Coomassie blue dye. The selected protein band from Elution was identified by MALDI-TOF-MS technology. **(C)** Immunoblotting with anti-γ-actin antibody. The protein fractions were resolved by gel electrophoresis and detected by Western blotting with anti-γ-actin antibody.

The SUMO2-modified proteins were isolated and identified through a multi-step procedure as outlined in Figure 1A. Firstly, a mammalian expression vector plasmid pcDNA3.1-N-Strep-SUMO2 was constructed for expressing N-terminal Strep-tagged SUMO2 fusion protein. After transfection with pcDNA3.1-N-Strep-SUMO2 and cultured for 48 h, H9c2 cells were lysed and subjected to affinity purification on Strep-Tactin agarose from IBA Lifesciences. Thirdly, a major portion (~90%) of the eluted proteins were resolved by gel electrophoresis and detected by Coomassie blue dye. As shown in Figure 1B, the elution showed a predominant protein band at the size of ~42 kDa. Fourthly, the protein band was identified by MALDI-TOF-MS technology. Peptide mapping suggested gamma actin (γ-actin) as the most likely candidate protein with the protein score and total ion score of 238 and 117, respectively. Finally, the minor portion (~10%) of the eluted proteins were resolved by gel electrophoresis and detected by Western blotting. As shown in Figure 1C, the anti-γ-actin antibody detected the SUMO2-bound protein with the size of ~42 kDa.

### SUMO2ylation of γ-actin was validated by confocal fluorescence microscopy and immunoprecipitation

**Figure 2:**
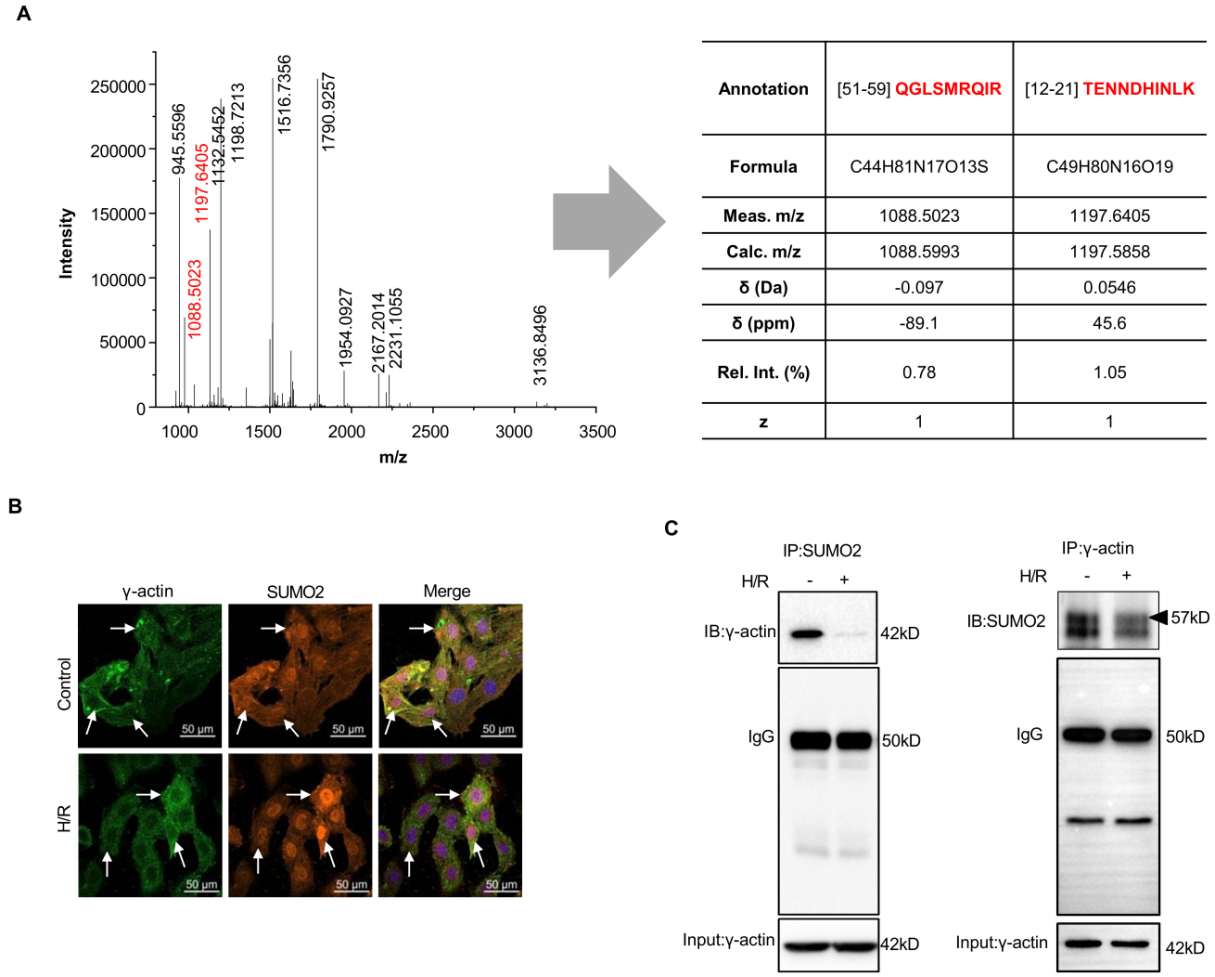
** Validation of the covalent SUMO2 modification of γ-actin. (A)** Search for SUMO2 peptides from the γ-actin protein band. The SUMO2 peptides were identified by MALDI*-*TOF*-*MS peptide mapping. The green dots indicated the peptide sequences derived from SUMO2. **(B)** Immunofluorescence imaging of intracellular SUMO2 and γ-actin. Following the challenge by sequential 3 h hypoxia and 24 h reoxygenation, H9c2 cells were immunostained with specific antibodies against SUMO2 and γ-actin and imaged under a confocal fluorescence microscope. The extent of colocalization was indexed by Pearson's coefficient (see Results). **(C)** Co-immunoprecipitation identification of covalent SUMO2-γ-actin conjugation. Following sequential hypoxia and reoxygenation, H9c2 cells were subjected to immunoprecipitation with anti-SUMO2 antibody or anti-γ-actin antibody and subsequent immunoblotting with anti-γ-actin antibody or anti-SUMO2 antibody. Arrow indicated the band at the size of 57 kD.

To validate the covalent interaction of SUMO2 with γ-actin, firstly, MS/MS peptide fragments were further analyzed for SUMO2-derived peptides by an open source mass spectrometry tool mMass (www.mmass.org) as previously described^39^, ^40^. As shown in Figure 2A, the selected protein band yielded two molecule ions at m/z 1088.5023 (z = +1) and 1197.6405 (z = +1) for two SUMO2-derived peptides with the sequences of TENNDHINLK and QGLSMRQIR. Secondly, the intracellular colocalization of SUMO2 and γ-actin was examined by immunofluorescence imaging and quantified by the Pearson correlation coefficient (γ) as previously described 41. Prior to fluorescence immunostaining and imaging, H9c2 cells were sequentially exposed to 3 h hypoxia and 24 h reoxygenation. As shown in Figure 2B, under normal culture conditions, γ-actin and SUMO2 were colocalized with the Pearson's coefficient value of γ= 0.8103 ± 0.016. Interestingly, hypoxia and reoxygenation reduced the colocalization of γ-actin and SUMO2 to the Pearson's coefficient value of γ= 0.6906 ± 0.013. Specifically, γ-actin was predominantly detected in the cytosol whereas SUMO2 was present in the cell nuclei. Thirdly, we performed co-immunoprecipitation to verify the SUMOylation of γ-actin. In practice, two parallel co-immunoprecipitation experiments were performed either with anti-SUMO2 antibody for pulldown and anti-γ-actin antibody for bloting or with anti-γ-actin antibody for pulldown and anti-SUMO2antibody for bloting. As shown in Figure 2C, γ-actin was isolated with anti-SUMO2 antibody from the cells under normal culture conditions but far less γ-actin was isolated from the cells challenged by hypoxia reoxygenation. On the other hand, pulldown with anti-γ-actin antibody resulted in strong and smear SUMO2 signals from the cells under normal culture conditions and a great increase in SUMO2 signals from the cells challenged by hypoxia reoxygenation. It is noteworthy that immunoprecipitation with anti-γ-actin antibody showed protein bands with molecular weight increase up to ~57 kD, suggesting the presence of SUMO2 modification.

### Myocardial infarction reduced the nuclear translocation of γ-actin and induced oxidative DNA damage

**Figure 3:**
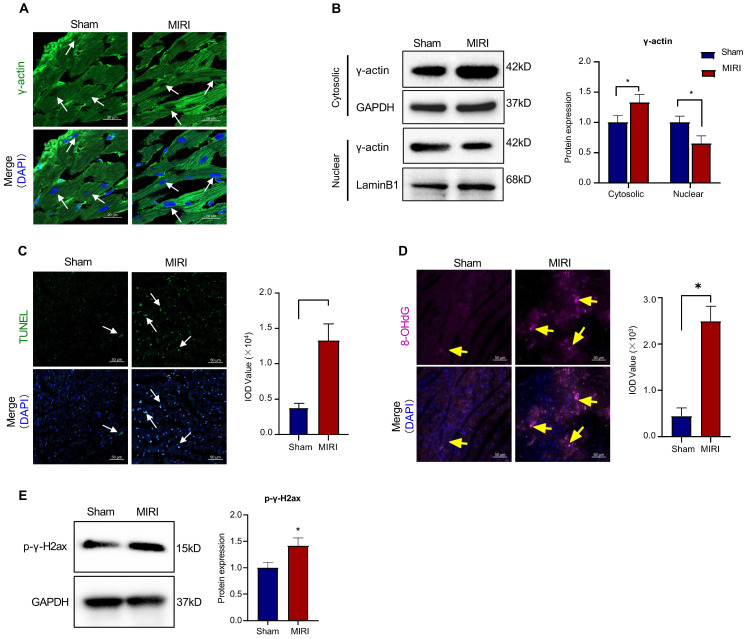
** Intracellular localization of γ-actin and DNA damage in a mouse model of myocardial infarction. (A)** Immunofluorescence imaging of intracellular γ-actin. Following the induction of myocardial infarction by LAD ligation, cardiac tissues from C57BL/6N mice were analyzed by immunofluorescence imaging with anti-γ-actin antibody. Scale bar: 20 µm. **(B)** Western blot nalysis of cytosolic and nuclearγ-actin. Following the induction of myocardial infarction by LAD ligation, cytosolic and nuclear proteins were isolated from the cardiac tissues and analyzed by Western blotting with anti-γ-actin antibody. The blots were quantified by a densitometric method. **(C)** TUNEL staining of cardiac tissues. Following the induction of myocardial infarction by LAD ligation, cardiac tissues were stained with TUNEL staining. The cell nuclei were stained with DAPI. The images were quantified by ImageJ software. Scale bar: 50 µm. **(D)** Immunostaining of 8-OHdG. Following the induction of myocardial infarction by LAD ligation, cardiac tissues were probed with anti-8-OHdG antibody and visualised with Alexa Fluor 647-conjugated secondary antibody. The cell nuclei were stained with DAPI. The images were quantified by ImageJ software. Scale bar: 50 µm. **(E)** Western blot analysis of p-γ-H2ax. Following the induction of myocardial infarction by LAD ligation, cardiac tissues were analyzed by Western blotting with anti-p-γ-H2ax antibody. The blots were quantified by a densitometric method. The results were shown as mean ± SD (n = 6), *p < 0.05.

To validate the functional correlationship of the nuclear γ-actin in myocardial infarction, we examined the nuclear localization of γ-actin in the mouse model of myocardial infarction. Firstly, the cardiac tissues were analyzed by immunofluorescence staining for the distribution of γ-actin. As shown in Figure 3A, myocardial ischemia reperfusion injury decreased the nuclear γ-actin in mice. Secondly, the cardiac tissues were lysed, separated into cytosolic and nuclear proteins, resolved by SDS-PAGE and anlyzed by Western blotting with anti-γ-actin antibody. As shown in Figure 3B, myocardial ischemia reperfusion injury increased the level of cytosolic γ-actin while decreased the level of nuclear γ-actin. Thirdly, the cardiac damage was examined by TUNEL assay. As shown in Figure 3C, myocardial ischemia reperfusion markedly increased the TUNEL positive cells. Fourthly, the oxidative DNA damage was examined by staining 8-OHdG. As shown in Figure 3D, myocardial ischemia reperfusion markedly increased the formation of 8-OHdG. Fifthly, the double-stranded DNA breaks was examined by staining p-γ-H2ax. As shown in Figure 3E, myocardial ischemia reperfusion markedly increased the level of p-γ-H2ax.

**Figure 4:**
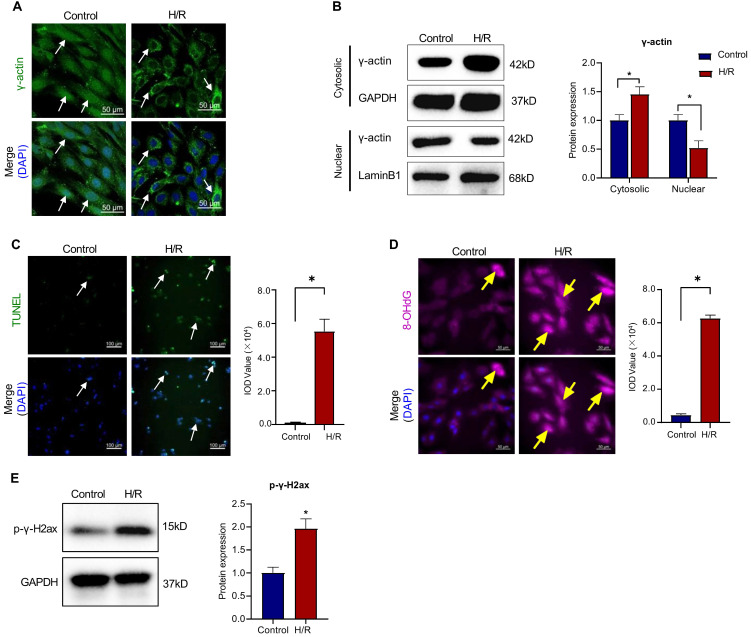
** Intracellular localization of γ-actin and DNA damage in H9c2 cell model. (A)** Immunofluorescence imaging of intracellular γ-actin. After exposure to 3 h hypoxia (0.1% O_2_) and 24 h reoxygenation (H/R), H9c2 cells were analyzed by immunofluorescence imaging with anti-γ-actin antibody. Scale bar: 50 µm. **(B)** Western blot nalysis of cytosolic and nuclear proteins. Following H/R treatment, cytosolic and nuclear proteins were isolated from H9c2 cells and analyzed by Western blotting with anti-γ-actin antibody. The blots were quantified by a densitometric method. **(C)** TUNEL staining of H9c2 cells. Following H/R treatment, H9c2 cells were stained with TUNEL staining. The cell nuclei were stained with DAPI. The images were quantified by ImageJ software. Scale bar: 100 µm. **(D)** Immunostaining of 8-OHdG. Following H/R treatment, H9c2 cells were probed with anti-8-OHdG antibody and visualised with Alexa Fluor 647-conjugated secondary antibody. The cell nuclei were stained with DAPI. The images were quantified by ImageJ software. Scale bar: 50 µm. **(E)** Western blot analysis of p-γ-H2ax. Following H/R treatment, H9c2 cells were analyzed by Western blotting with anti-p-γ-H2ax antibody. The blots were quantified by a densitometric method. The results were shown as mean ± SD (n = 3), *p < 0.05.

To validate the functional correlationship of the nuclear γ-actin in the *in vitro* H9c2 cell model, following sequential challeng by hypoxia for 3 h and reoxygenation for 24 h, we performed immunofluorescence staining and Western blot analysis for the intracellular localization of γ-actin, TUNEL assay for the cardiac damage, 8-OHdG staining for the oxidative DNA damage and p-γ-H2ax staining for the double-stranded DNA breaks. As shown in Figure 4A-E, *in vitro* results were basically consistent with the corresponding results from the mouse model of myocardial infarction. These results consolidated that ischemia reperfusion reduced the level of nuclear γ-actin, initiated oxidative DNA damage and induced apoptosis.

### SUMOylation of γ-actin by SUMO2 promoted the nuclear translocation of γ-actin and reduced DNA damage against myocardial infarction injury

**Figure 5:**
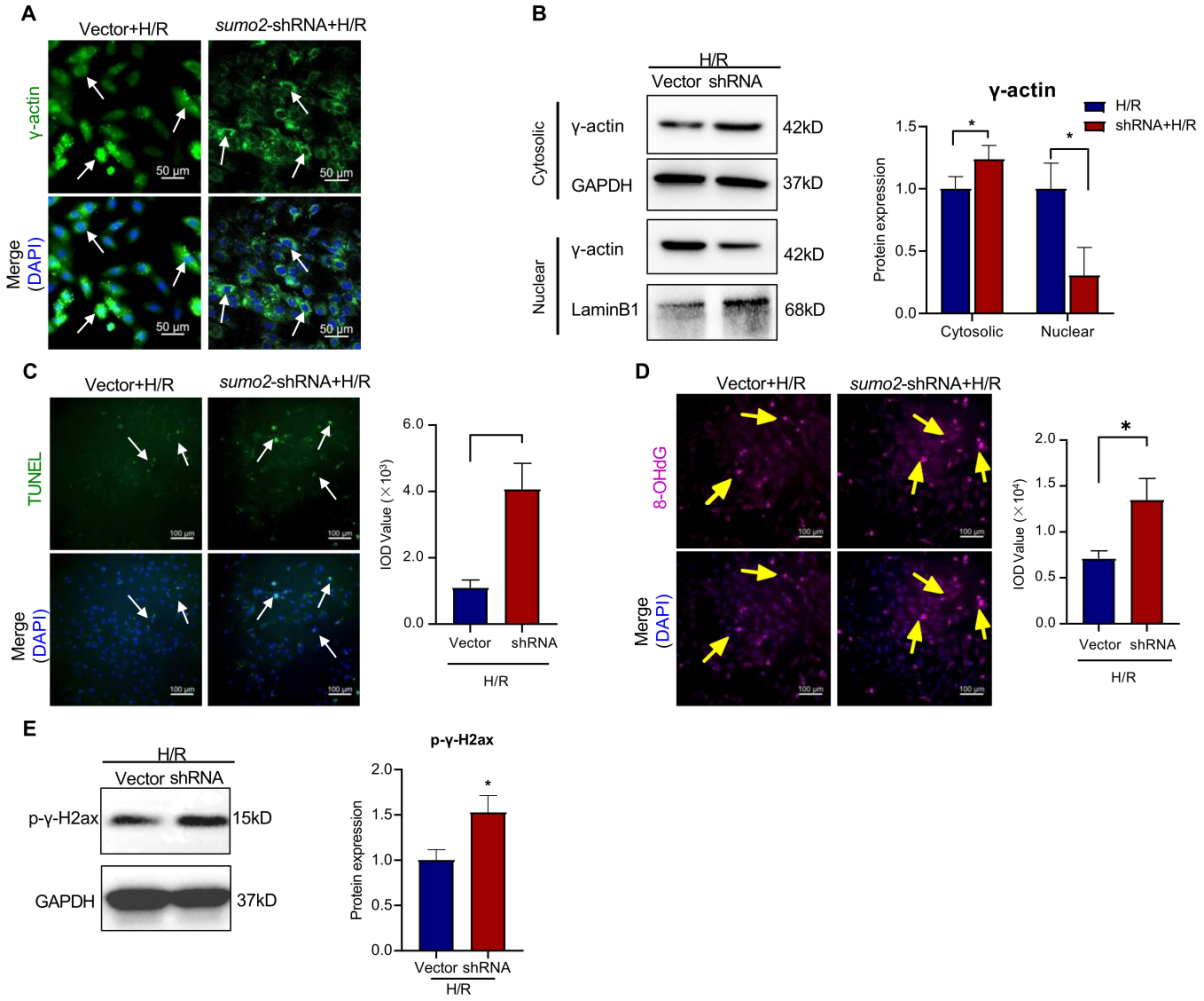
** Effects of SUMO silencing on the nuclear translocation of γ-actin and DNA damage in H9c2 cell model.** SUMO2 was silenced by introducing lentiviral SUMO2 shRNA into H9c2 cells while the lentiviral vector was used as the control. **(A)** Immunofluorescence imaging of intracellular γ-actin. After exposure to 3 h hypoxia (0.1% O_2_) and 24 h reoxygenation (H/R), the transfected H9c2 cells were analyzed by immunofluorescence imaging with anti-γ-actin antibody. Scale bar: 50 µm. **(B)** Western blot nalysis of cytosolic and nuclear proteins. Following H/R treatment, cytosolic and nuclear proteins were isolated from the transfected H9c2 cells and analyzed by Western blotting with anti-γ-actin antibody. The blots were quantified by a densitometric method. **(C)** TUNEL staining of the transfected H9c2 cells. Following H/R treatment, H9c2 cells were stained with TUNEL staining. The cell nuclei were stained with DAPI. The images were quantified by ImageJ software. Scale bar: 100 µm. **(D)** Immunostaining of 8-OHdG. Following H/R treatment, the transfected H9c2 cells were probed with anti-8-OHdG antibody and visualised with Alexa Fluor 647-conjugated secondary antibody. The cell nuclei were stained with DAPI. The images were quantified by ImageJ software. Scale bar: 100 µm. **(E)** Western blot analysis of p-γ-H2ax. Following H/R treatment, the transfected H9c2 cells were analyzed by Western blotting with anti-p-γ-H2ax antibody. The blots were quantified by a densitometric method. The results were shown as mean ± SD (n = 3), *p < 0.05.

To clarify the effects of SUMO2 expression on the SUMOylation and nuclear translocation of γ-actin and the DNA damage in cardiomyocytes, we performed lentivirus-mediated knockdown and overexpression of SUMO2 in H9c2 cells. The tranfected cells were challenged by hypoxia and reoxygenation. SUMO2 knockdown cells were initially examined for the intracellular localization of γ-actin by immunofluorescence staining and Western blot analysis, the cardiac damage by TUNEL assay, the oxidative DNA damage by 8-OHdG staining and the double-stranded DNA breaks by p-γ-H2ax staining. As shown in Figure 5A-E, SUMO2 knockdown decreased the levels of nuclear γ-actin while increased the number of TUNEL positive cells, the levels of 8-OHdG and p-γ-H2ax (*p<0.05).

**Figure 6:**
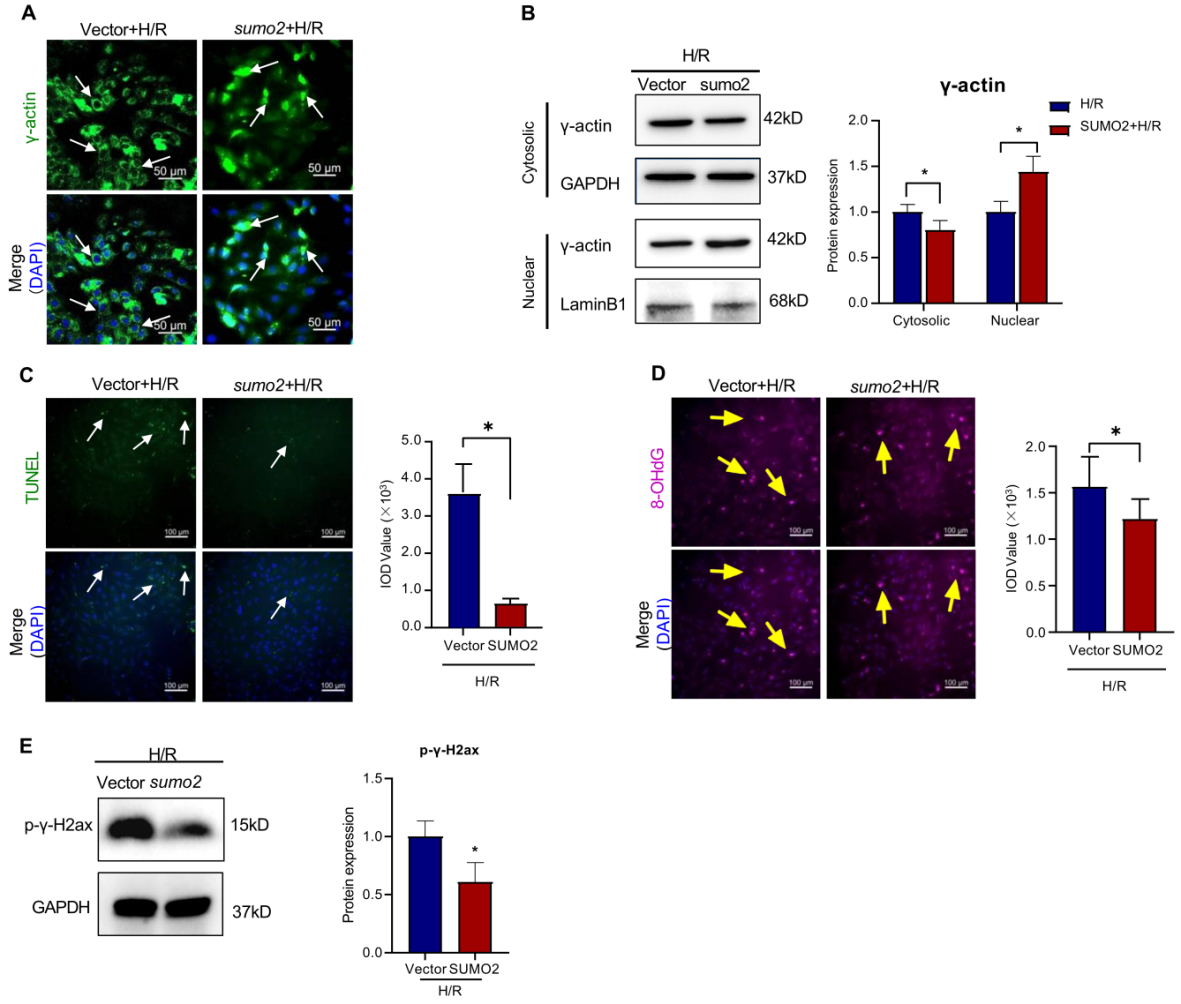
** Effects of SUMO overexpression on the nuclear translocation of γ-actin and DNA damage in H9c2 cell model.** SUMO2 was silenced by introducing lentiviral SUMO2 cDNA into H9c2 cells while the lentiviral vector was used as the control. **(A)** Immunofluorescence imaging of intracellular γ-actin. After exposure to 3 h hypoxia (0.1% O_2_) and 24 h reoxygenation (H/R), the transfected H9c2 cells were analyzed by immunofluorescence imaging with anti-γ-actin antibody. Scale bar: 50 µm. **(B)** Western blot nalysis of cytosolic and nuclear proteins. Following H/R treatment, cytosolic and nuclear proteins were isolated from the transfected H9c2 cells and analyzed by Western blotting with anti-γ-actin antibody. The blots were quantified by a densitometric method. **(C)** TUNEL staining of the transfected H9c2 cells. Following H/R treatment, H9c2 cells were stained with TUNEL staining. The cell nuclei were stained with DAPI. The images were quantified by ImageJ software. Scale bar: 100 µm. **(D)** Immunostaining of 8-OHdG. Following H/R treatment, the transfected H9c2 cells were probed with anti-8-OHdG antibody and visualised with Alexa Fluor 647-conjugated secondary antibody. The cell nuclei were stained with DAPI. The images were quantified by ImageJ software. Scale bar: 100 µm. **(E)** Western blot analysis of p-γ-H2ax. Following H/R treatment, the transfected H9c2 cells were analyzed by Western blotting with anti-p-γ-H2ax antibody. The blots were quantified by a densitometric method. The results were shown as mean ± SD (n = 3), *p < 0.05.

On the other hand, SUMO2-overexpressing cells were examined for the intracellular localization of γ-actin by immunofluorescence staining and Western blot analysis, the cardiac damage by TUNEL assay, the oxidative DNA damage by 8-OHdG staining and the double-stranded DNA breaks by p-γ-H2ax staining. As shown in Figure 6A-E, SUMO2 overexpression increased the levels of nuclear γ-actin while decreased the number of TUNEL positive cells, the levels of 8-OHdG and p-γ-H2ax (*p<0.05).

**Figure 7:**
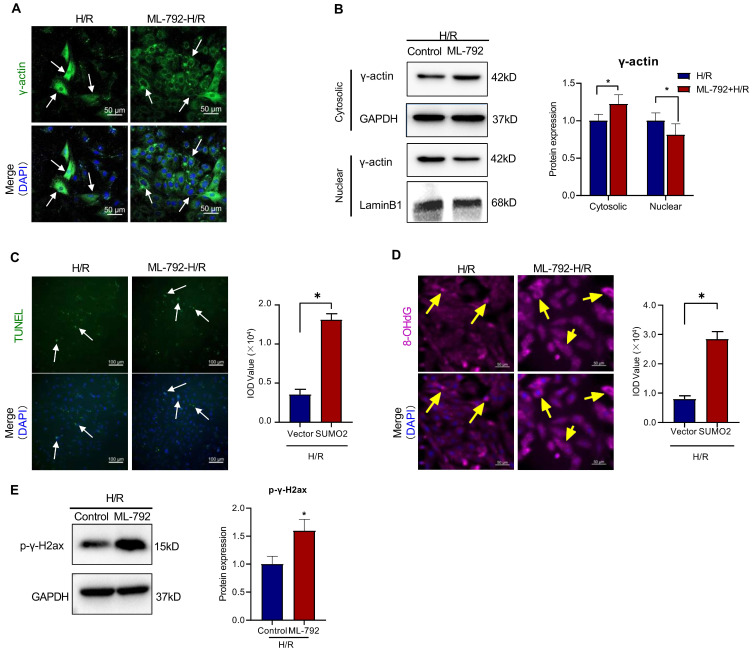
** Effects of SUMOylation inhibitor ML-792 on the nuclear translocation of γ-actin and DNA damage in H9c2 cell model.** H9c2 cells were pre-treated with ML-792 or vehicle and subsequently challenged by H/R treatment. the lentiviral vector was used as the control. **(A)** Immunofluorescence imaging of intracellular γ-actin. H9c2 cells were analyzed by immunofluorescence imaging with anti-γ-actin antibody. Scale bar: 50 µm. **(B)** Western blot analysis of cytosolic and nuclear proteins. Cytosolic and nuclear proteins were isolated from H9c2 cells and analyzed by Western blotting with anti-γ-actin antibody. The blots were quantified by a densitometric method. **(C)** TUNEL staining of H9c2 cells. H9c2 cells were stained with TUNEL staining. The cell nuclei were stained with DAPI. The images were quantified by ImageJ software. Scale bar: 100 µm. **(D)** Immunostaining of 8-OHdG. H9c2 cells were probed with anti-8-OHdG antibody and visualised with Alexa Fluor 647-conjugated secondary antibody. The cell nuclei were stained with DAPI. The images were quantified by ImageJ software. Scale bar: 50 µm. **(E)** Western blot analysis of p-γ-H2ax. H9c2 cells were analyzed by Western blotting with anti-p-γ-H2ax antibody. The blots were quantified by a densitometric method. The results were shown as mean ± SD (n = 3), *p < 0.05.

Furthermore, SUMOylation inhibitor ML-792 was evaluated for inhibiting SUMOylation and the nuclear translocation of γ-actin in the *in vitro* H9c2 cell model. As shown in Figure 7A-B, ML-792 effectively descreased the level of nuclear γ-actin while increased the level of cytosolic γ-actin. As shown in Figure 7 C-E, consistently, ML-792 markedly reduced the number of TUNEL positive cells and the formation of 8-OHdG and p-γ-H2ax (*p<0.05).

### The SUMOylation sites K^68^ and K^284^ are essential for the nuclear translocation of γ-actin and DNA damage repair against myocardial infarction injury

**Figure 8:**
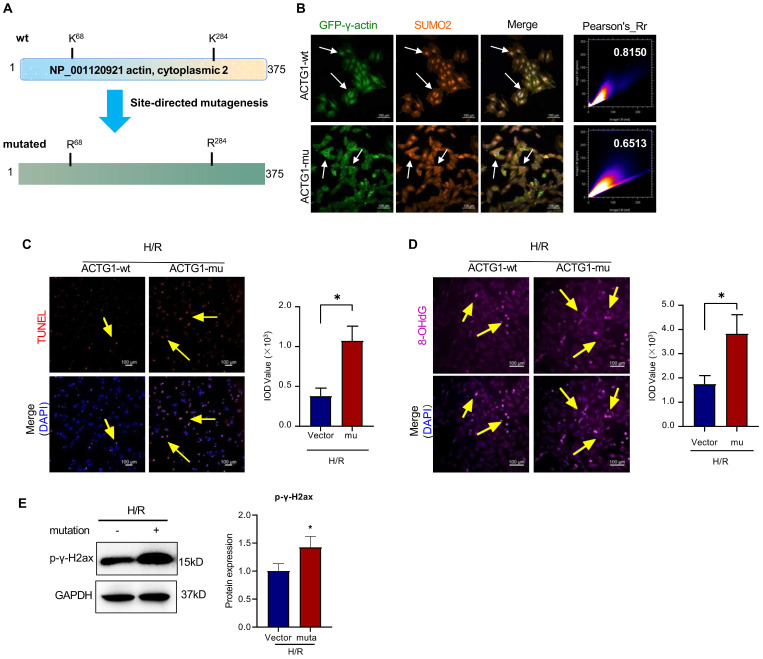
** Site-directed mutagenesis of SUMOylation sites (i.e., K^68^, K^284^) and subsequent impact on the nuclear translocation of γ-actin and DNA damage in H9c2 cell model. (A)** Prediction and site-directed mutagenesis of SUMOylation sites in γ-actin. The lysine residues K^68^ and K^284^ in γ-actin were predicted as SUMOylation sites by SUMOplot Analysis program (https://www.abcepta.com/sumoplot). Lentiral plasmid pCDH-CMV-ACTG-wt for wild type γ-actin was converted to pCDH-CMV-ACTG-mu for mutated γ-actin (K^68^R/K^284^R) by site-directed mutagenesis. **(B)** Immunofluorescence imaging of intracellular γ-actin and SUMO2. H9c2 cells were transiently tranfected with pdsRED-SUMO2 with pEGFP-ACTG-wt or pEGFP-ACTG-mu. After H/R treatment, the transfected H9c2 cells were analyzed by immunofluorescence imaging for EGFP and dsRED. Scale bar: 50 µm. **(C)** TUNEL staining of the transfected H9c2 cells. pCDH-CMV-ACTG-wt and pCDH-CMV-ACTG-mu were incorporated into the corresponding lentirus particles for infecting H9c2 cells. and After H/R challenge, stable H9c2 cells (i.e, ACTG1-wt, ACTG1-mu) were stained with TUNEL staining. The cell nuclei were stained with DAPI. The images were quantified by ImageJ software. Scale bar: 100 µm. **(D)** Immunostaining of 8-OHdG. After H/R challenge, stable H9c2 cells (i.e, ACTG1-wt, ACTG1-mu) were probed with anti-8-OHdG antibody and visualised with Alexa Fluor 647-conjugated secondary antibody. The cell nuclei were stained with DAPI. The images were quantified by ImageJ software. Scale bar: 100 µm. **(E)** Western blot analysis of p-γ-H2ax. After H/R challenge, stable H9c2 cells (i.e, ACTG1-wt, ACTG1-mu) were analyzed by Western blotting with anti-p-γ-H2ax antibody. The blots were quantified by a densitometric method. The results were shown as mean ± SD (n = 3), *p < 0.05.

To determine the effects of SUMOylation on the biological functions of γ-actin, firstly, we searched for the potential SUMOylation sites in γ-actin peptide sequence using the SUMOplot Analysis program provided by Abgent. As shown in Figure 8A, we identified two critical lysine residues (i.e., K^68^ and K^284^) for SUMOylation. Secondly, we cloned the full length γ-actin cDNA into lentiviral expression vector to yield plasmid construct pCDH-CMV-ACTG and performed subsequent point mutations to yield plasmid construct pCDH-CMV-ACTG (K^68^R/K^284^R). Plasmid constructs pCDH-CMV-ACTG and pCDH-CMV-ACTG (K^68^R/K^284^R) were packaged into lentiviral particles for infecting H9c2 cells and preparing stable clones. Thirdly, H9c2 cells overexpressing intact γ-actin or γ-actin (K^68^R/K^284^R) were examined for the intracellular localization of γ-actin by immunofluorescence staining and Western blot analysis, the cardiac damage by TUNEL assay, the oxidative DNA damage by 8-OHdG staining and the double-stranded DNA breaks by p-γ-H2ax staining. As shown in Figure 8B, following transient cotransfection of pDsRED-SUMO2 with either pEGFP-N1-ACTG for intact γ-actin or pEGFP-N1-ACTG (A^203^G/A^851^G) for mutated γ-actin (K^68^R/K^284^R), H9c2 cells were examined for the cellular colocalisation of SUMO2 and γ-actin by fluorescence imaging. Interestingly, SUMO2 and mutated γ-actin (K^68^R/K^284^R) were far less colocalised in H9c2 cells with Pearsons coefficient of 0.6906 ± 0.013 compared with that with intact γ-actin with Pearsons coefficient of 0.8103 ± 0.016. As for the biological impact of γ-actin SUMOylation sites (K^68^/K^286^), as shown in Figure 8C-E, H9c2 cells overexpressing γ-actin (K^68^R/K^284^R) showed markedly increase in the TUNEL positive cells and the formation of 8-OHdG and p-γ-H2ax compared with the cells overexpressing intact γ-actin (*p<0.05).

## Discussion

SUMOylation of proteins has emerged as an important therapeutic target for drug discovery 8, 14. We recently demonstrated that natural product puerarin protected cardiomyocytes against ischemia reperfusion injury via up-regulating SUMO2 and SUMOylation without altering SUMO1 expression 8. The present study was designed to identify SUMO2-modified proteins and elucidate the cardioprotective mechanisms. SUMOylated proteins were successfully isolated by several strategies including SUMO antibody, an N-terminal tandem affinity protein tag, N-6xHis-SUMO transfection and GST-SIM fusion protein 42-44. In the present study, we introduced the plasmid construct pcDNA3.1-N-Strep-SUMO2 into H9c2 cells to overexpress a N-Strep-SUMO2 fusion protein. Upon SUMOylation by N-Strep-SUMO2, the SUMOylated proteins were isolated by Strep-Tactin XT 4Flow resin as outlined in Figure 1A. Interestingly, γ-actin was isolated as a major SUMO2ylated protein (Figure 1B & 1C). Therefore, this study investigated whether and how SUMOylation of γ-actin could be a new cardioprotective mechanism.

γ-Actin is encoded by the ACTG1 gene, widely expressed in many tissues and well recognized as a key component of the cellular cytoskeletons for regulating a wide range of cellular activities including cell motility, cell shape maintenance, polarity, chemotaxis, endocytosis and phagocytosis 45, 46. Increasing evidence indicates that γ-actin plays ermerging roles in the cell nuclear processes including chromatin remodeling, transcription, replication and DNA repair, representing a new frontier in cell biology 47-49. The newly synthesized monomeric γ-actin is transported into the cell nucleus via importin-9 and regulated to form the dynamic equilibrium between monomeric globular form (G-form) and polymerised filamentous form (F-form) 50. Nuclear F-actin appeared to highly associated with DNA repair sites, possibly through interactions with different actin-binding proteins including DNA damage and repair factors 51, 52. Dysregulation of nuclear actin dynamics and impairment of DNA repair were detected in lymphocytes and Huntington's disease (HD) 53, 54. Presumably, the functional diversity of γ-actin is tightly regulated by various PTMs including acetylation, methylation, oxidation, phosphorylation and sumoylation 55, 56. DNA damage is implicated in the pathology of myocardial infarction and other heart diseases 57. Timely intitiation of DNA repair is crucial to maintain genome integrity and enhance survival 58. On the other hand, we recently proposed that SUMO2 might exhibit cardioprotective activity via increasing SUMOylation of specific proteins in myocardial infarction 8. In the present study, firstly, we validated that the discrepancy in the intracellular localization of SUMO2 and γ-actin and the status of DNA damage in a mouse model of myocardial infarction and H9c2 cell model of hypoxia reoxygenation. Our results showed that less nuclear γ-actin was correlated to more DNA damage (Figure 3 & Figure 4). Secondly, we determined the relationship between the expression level of SUMO2 and the nuclear localization of γ-actin as well as the impact of SUMO2 on DNA damage. Through silencing or overexpressing SUMO2 in H9c2 cell model, we found that SUMO2 expression supported the nuclear translocation of γ-actin and subsequently reduced DNA damage (Figure 5 & Figure 6). Thirdly, we examined the effects of SUMOylation inhibitor ML-792 on the colocalization of SUMO2 and γ-actin in the cell nucleus and the extent of DNA damage. Our results showed that SUMOylation inhibition markedly reduced the nuclear level of γ-actin and increased the extent of DNA damage (Figure 7). Despite the elusiveness of the mechanism by which nuclear γ-actin promotes DNA repair 50, our results consolidated an essential role for nuclear γ-actin in the control of DNA damage and also demonstrated the positive impact of SUMO2 expression on the participation of γ-actin in DNA repair.

SUMO modification appears to be a key PTM for regulating the nuclear import of target proteins 59. Chen L et al. reported that SUMOylation primarily modified the consensus lysine residue K^284^ in human ZIC3 and increased the nuclear retention of ZIC3 60. SUMOylation promoted the nuclear localization of S100A10 in polyploid tumor giant cells and their daughter cells for stimulating the proliferation and migration of cells 61. Hofmann WA et al performed computational prediction and site-mutagenesis to reveal that the lysine residues K^68^ and K^284^ in actin were covalently modified by SUMO2 and SUMO3 and demonstrated that SUMOylation was required for the nuclear localization of actin 62. In the present study, firstly, we searched for the SUMOylation sites in the peptide sequence of γ-actin (Accession NP_001120921) at the online GPS-SUMO platform (http://sumosp.biocuckoo.org/). As a result, GPS-SUMO predicted two SUMOylation sites: K^68^ with the score of 2.618 and p value of 0.982 and K^284^ with the score of 3.696 and p value of 0.039 (Figure 8A). We further validated that the lysine residues K^68^ and K^284^ were two high probability consensus SUMOylation sites with the SUMOplot Analysis program (https://www.abcepta.com/sumoplot). Secondly, we clarified whether the lysine residues K^68^ and K^284^ were required for SUMOylation and the nuclear translocalization of γ-actin. As for the selection of amino acid residues for mutagenesis, Alonso A et al. approved that the mutagenesis of two lysine residues to arginine residues well preserved the structural integrity of γ-actin 63. Thus, we also performed site-directed mutagenesis to change two lysine residues K^68^ and K^284^ into two arginine residues R^68^ and R^284^ and generated two stable H9c2 cell lines expressing intact γ-actin and mutated γ-actin, respectively (Figure 8A). Thirdly, we performed transient co-overexpression of DsRED-SUMO2 with either EGFP-γ-actin or EGFP-γ-actin (K^68^R/K^284^R) in H9c2 cells and examined the intracellular localization of EGFP and DsRED. Our results showed that mutated γ-actin (K^68^R/K^284^R) no longer colocalized with SUMO2 in the cell nucleus, suggesting the essential roles of two lysine residues K^68^ and K^284^ for SUMOylation and nuclear localization of γ-actin. Fourthly, we examined the liability of two stable H9c2 cell lines to hypoxia reoxygenation challenge. Our results showed that the stable H9c2 cell line overexpressing mutated γ-actin (K^68^R/K^284^R) were more liable to hypoxia reoxygenation injury (Figure 8C-E), suggesting the protective role for SUMOylation of γ-actin by SUMO2. However, the future study should be designed to clarify: 1) which of monomeric or polymeric γ-actin is SUMOylated by SUMO2; 2) where (i.e., cytosol or nucleus) γ-actin is SUMOylated by SUMO2; 3) how SUMO2-mediated SUMOylation affects the function of nuclear γ-actin.

## Conclusion

In the present study, we successfully isolated γ-actin as a major SUMOylated protein from rat cardiomyocyte cell line H9c2 through overexpressing a N-Strep-SUMO2 fusion protein. SUMO2 expression appeared to control the colocalization SUMO2 and γ-actin in the cell nucleus and the extent of DNA damage in the cells exposed to ischemia reperfusion injury. Two lysine residues K68 and K284 were proven to be essential for the SUMOylation and nuclear translocation of γ-actin. Overall, our results suggested that the SUMOylation of γ-actin by SUMO2 might be a new cardioprotective mechanism against myocardial ischemia reperfusion injury.

## Abbreviations

SUMO2: Small Ubiquitin-like Modifier 2
TUNEL: terminal deoxynucleotidyl transferase dUTP nick end labeling
MI: myocardial infarction
PTMs: post-translational modifications
DMEM: Dulbecco's modified Eagle's medium
LAD: left anterior descending artery
DAPI: 4′,6-diamidino-2-phenylindole
FBS: fetal bovine serum
H/R: hypoxia/reoxygenation
ACTG: actin, cytoplasmic 2
GAPDH: glyceraldehyde-3-phosphate dehydrogenase
MEK: mitogen-activated protein kinase
ERK: extracellular signal-regulated kinase
PI3K: phosphatidylinositol 3-kinase
N-Strep-SUMO2: N-terminal Strep-tagged SUMO2 fusion protein
